# Gα_i1_ inhibition mechanism of ATP-bound adenylyl cyclase type 5

**DOI:** 10.1371/journal.pone.0245197

**Published:** 2021-01-25

**Authors:** Daniele Narzi, Siri C. van Keulen, Ursula Röthlisberger

**Affiliations:** Institut des Sciences et Ingénierie Chimiques, École Polytechnicque Fédérale de Lausanne (EPFL), Lausanne, Switzerland; University of Queensland, AUSTRALIA

## Abstract

Conversion of adenosine triphosphate (ATP) to the second messenger cyclic adenosine monophosphate (cAMP) is an essential reaction mechanism that takes place in eukaryotes, triggering a variety of signal transduction pathways. ATP conversion is catalyzed by the enzyme adenylyl cyclase (AC), which can be regulated by binding inhibitory, G*α*_*i*_, and stimulatory, G*α*_*s*_ subunits. In the past twenty years, several crystal structures of AC in isolated form and complexed to G*α*_*s*_ subunits have been resolved. Nevertheless, the molecular basis of the inhibition mechanism of AC, induced by G*α*_*i*_, is still far from being fully understood. Here, classical molecular dynamics simulations of the isolated *holo* AC protein type 5 and the *holo* binary complex AC5:G*α*_*i*_ have been analyzed to investigate the conformational impact of G*α*_*i*_ association on ATP-bound AC5. The results show that G*α*_*i*_ appears to inhibit the activity of AC5 by preventing the formation of a reactive ATP conformation.

## Introduction

The nucleotide adenosine triphosphate (ATP) plays a crucial role in metabolism, not only as primary energy carrier, but also in intracellular signal transduction, acting as a substrate for the formation of the second messenger cyclic adenosine monophosphate (cAMP). cAMP is produced through deprotonation and cyclization of ATP, catalyzed by the enzyme adenylyl cyclase (AC) [1, 2]. Nine different membrane-bound isoforms of AC are known in nature, ranging from AC1 to AC9 [3]. Structurally, such isoforms consist of two membrane regions, M1 and M2, and two cytoplasmatic domains, C1 and C2, of which the C1/C2 interface harbours the enzyme’s active site. Once cAMP dissociates from the C1/C2 interface, the second messenger is able to transduce a signal in the cell by, for example, activating protein kinase A and affecting ion channels located in the lipid bilayer [4–6]. Apart from their primary function as signal transducers, ACs may also work as signal integrators since these enzymes can act as decision functions, determining the amount and the time of cAMP release. A typical decision function, characteristic for several AC enzymes, is the detection of co-occuring signaling events, also known as coincidence detection [7–13], which is known to be involved in memory and reward learning mechanisms [14–16].

**Fig 1:**
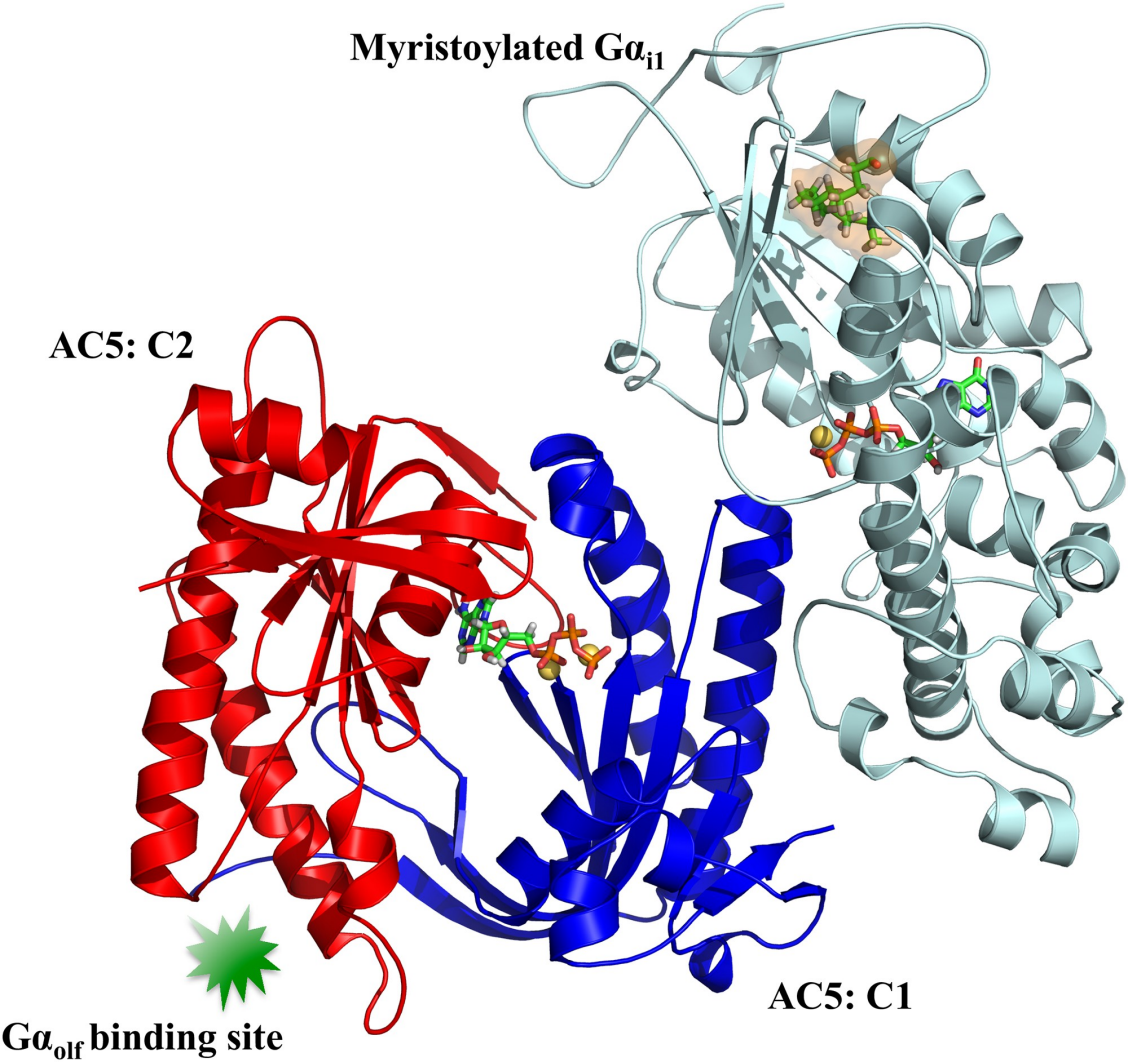
Simulated system. The binary complex consisting of AC5’s catalytic domains C1 (blue) and C2 (red), bound to the G*α*_*i*1_ subunit (cyan) is shown in cartoon representation. ATP and GTP molecules as well as the myristoyl group are represented as sticks. Mg^2+^ ions are depicted as yellow spheres. The binding site for G*α*_*olf*_ located on the C2 domain of AC5 is also highlighted.

cAMP regulation is governed by G-protein-coupled receptors (GPCRs). These receptors can activate inhibitory or stimulatory G proteins in the cytosol to enable or disable AC’s catalytic function. When G proteins are activated by GPCRs, their heterotrimeric structure, consisting of a *α*, *β* and *γ* subunit, dissociates, which results in a monomeric G*α* subunit and a *βγ* dimer [17]. Once a G*α* subunit is active, it can bind to AC isoforms via non-competitive association. Inhibitory G*α* (G*α*_*i*_) can interact with AC1, AC5 and AC6 via its C1 domain while stimulatory G*α* (G*α*_*s*_ or G*α*_*olf*_) can bind to all isoforms of membrane-bound AC through C2 association (Fig 1) [18–20].

Several X-ray structures have been resolved for the stimulated binary complex, AC:G*α*_*s*_ [21–23]. The majority of these AC X-ray structures only include the cytoplasmatic C1 and C2 domains. Nonetheless, these structures can be used to obtain a better understanding of the stimulatory effect of G*α*_*s*_ on AC5 as the cytoplasmatic domains are known to remain catalytically active even in the absence of the membrane-embedded protein regions [18, 24]. In contrast to G*α*_*s*_, no X-ray structures are available for the AC:G*α*_*i*_ dimer as crystallization of the myristoylated form of G*α*_*i*_ is challenging [25].

Recently, classical molecular dynamics (MD) simulations have been employed to gain insight into G*α*_*i*1_’s inhibitory mechanism by simulating an AC5:G*α*_*i*1_ and a G*α*_*olf*_:AC5:G*α*_*i*1_ complex in the absence of ATP [26, 27], i.e. with the *apo* form of AC5. The results of these studies show that the *apo* dimer and trimer are stable and indicate that the G*α*_*i*1_-bound AC5 structure undergoes conformational changes that lead to an inhibited state of the enzyme [26]. In particular, such conformational changes, occurring in both AC5:G*α*_*i*1_ and G*α*_*olf*_:AC5:G*α*_*i*1_ complexes, result in a reduced accessibility of the active site, thus decreasing the probability of substrate binding and therefore their catalytic activity. However, the presence of ATP could potentially affect G*α*_*i*1_’s inhibitory function as the substrate has a stimulatory effect on the catalytic function of AC5. Frezza *et al*., for instance, showed that the presence of ATP in the binding site of AC5 impacts the structure and flexibility of AC5 in both the isolated and G*α*_*s*_-bound state [28]. Therefore, the conformation and activity of AC5 in the AC5:G*α*_*i*1_ binary complex could potentially be influenced by the presence of ATP at the C1/C2 interface.

Here, molecular dynamics simulations are presented of the ATP-bound AC5:G*α*_*i*1_ binary complex and the *holo* G*α*-free AC5 protein. Conformations sampled by the protein and ATP along 3 *μ*s of simulation have been analyzed and compared between the two simulated systems. Additionally, a second simulation of the ATP-bound AC5:G*α*_*i*1_ binary complex was carried out for 700 ns in order to check the reproducibility of the results presented herein. The results show that the interactions between *holo* AC5 and G*α*_*i*1_ lead to a perturbation of the AC5 binding region for the stimulatory G*α*_*s*_ subunit, which suggests that the probability for G*α*_*s*_ binding is reduced. Additionally, in contrast with the *holo* G*α*-free AC5 protein, the conformations sampled by ATP in the *holo* AC5:G*α*_*i*1_ binary complex result in a non-reactive ATP state. These two conformational changes, occurring upon G*α*_*i*1_ association, are suggested to be at the basis of the inhibition mechanism of AC5 mediated by G*α*_*i*1_ binding.

## Materials and methods

### Initial structures

The crystal structure (PDB ID 1CJK) of AC(ATP*α*S):G*α*_*s*_ was used as a template for the *holo* binary AC5:G*α*_*i*1_ complex and the *holo* G*α*-free AC5 system [22]. This X-ray structure includes the catalytic AC domains, C1a and C2a, bound to an ATP analogue ATP*α*S (Adenosine-5’-rp-alpha-thio-triphosphate), and a G*α*_*s*_ subunit interacting with the catalytic domains of AC. The template used for modelling the active rat AC5 (UniprotKB Q04400) conformation in the binary complex consists of the C1 and C2 domains (C1a from canine AC5 and C2a from rat AC2) from 1CJK [22]. The G*α*_*i*1_ subunit was taken from reference [29], a modelled structure of the myristoylated Rattus norvegicus G*α*_*i*1_ subunit (UniprotKB P10824) interacting with guanosine-5’-triphosphate (GTP) and a Mg^2+^ ion. The active myristoylated Rattus norvegicus G*α*_*i*1_ is referred to as G*α*_*i*1_ because only a myristoylated form of G*α*_*i*1_ was used in the simulations. Myristoylation, crucial for G*α*_*i*1_’s inhibitory function [18, 19], is a post-translational modification of the N-terminus of G*α*_*i*1_ that results in the covalent attachment of a 14-carbon saturated fatty acid to the N-terminal glycine residue of G*α*_*i*1_ via an amide bond. The modelled AC5 and G*α*_*i*1_ structures were used for docking G*α*_*i*1_ on AC5’s C1 domain to obtain the initial binary complex conformation for simulation.

The HADDOCK web server [30] was used for docking ten conformations of the active G*α*_*i*1_ subunit to AC5’s catalytic domains in the *holo* form as described in references [26, 29]. The active region of G*α*_*i*1_ was defined in HADDOCK as a large part of the alpha helical domain (residues 112-167), the switch I region (residues 175-189) and the switch II region (residues 200-220), allowing for a large area on the G*α*_*i*1_ subunit surface to be taken into account during docking. The active region of AC5’s C1 domain was defined as the *α*1 helix (residues 479-490) and the C-terminal region of the *α*3 helix (residues 554-561) based on results from gel filtration that show that G*α*_*i*1_ is unable to interact with C2 and mutagenesis experiments that have confirmed that G*α*_*i*1_’s main interactions with AC is via the C1 domain [18]. Ten snapshots of G*α*_*i*1_ were used for docking the G*α* subunit to the catalytic domain of AC5. These snapshots were extracted at time intervals of 0.5 ns from the end of the classical MD trajectory of G*α*_*i*1_ (around 1.9 *μ*s) described in reference [29]. The same three criteria for complex selection as in reference [26] were applied: (1) the absence of overlap between the C2 domain and G*α*_*i*1_, (2) no overlap with the GTP binding region of G*α*_*i*1_ and the C1 domain of AC5, and (3) presence of similar complexes in the top-ten docking results of the docking calculations performed for all ten used G*α*_*i*1_ conformations. This last criterium increases the probability that the docked orientation of the selected complex is robust as similar AC5:G*α*_*i*1_ complexes can be obtained using different conformations of G*α*_*i*1_. Besides meeting these criteria, the orientation of G*α*_*i*1_ with respect to the membrane (absent in the simulation) was assessed to select a binary complex that would be consistent with a membrane environment. Moreover, the position of G*α*_*i*1_ on the C1 domain was required to be similar to its position on C1 in the absence of ATP [26].

### Classical molecular dynamics simulations

*Holo* G*α*-free AC5 and the *holo* AC5:G*α*_*i*1_ complex were solvated with ∼31 000 and ∼53 000 water molecules respectively. K^+^ and Cl^−^ ions were added to the system to obtain a physiological concentration of 150 mM. An excess of K^+^ ions was used to neutralize the system. The force fields used for the protein and the water molecules are AMBER99SB [31] and TIP3P [32], which were employed by Gromacs 5.1.2 [33, 34] to perform the simulations. The adjusted force field parameters for the Cl^−^ and K^+^ ions were taken from Joung *et al*. [35]. For GTP and ATP, the force field parameters generated by Meagher *et al*. were used [36]. The Mg^2+^ ion parameters originated from Allnér *et al*. [37] and the parameter set for the myristoyl group was taken from reference [29]. Both the *holo* G*α*-free AC5 and the *holo* AC5:G*α*_*i*1_ complex were simulated for 3 *μ*s at 310 K and 1 bar using a Nosé-Hoover thermostat [38, 39] and an isotropic Parrinello-Rahman barostat [40]. A replicate of the *holo* AC5:G*α*_*i*1_ system with different initial velocities was simulated for ∼0.7 *μ*s. Electrostatic interactions were calculated with the Ewald particle mesh method [41] with a real space cutoff of 12 Å. Bonds involving hydrogen atoms were constrained using the LINCS algorithm [42]. The integration time step was set to 2 fs. Images of both protein systems were prepared with Pymol [43].

## Results and discussion

### Stability and conformational analysis

**Fig 2:**
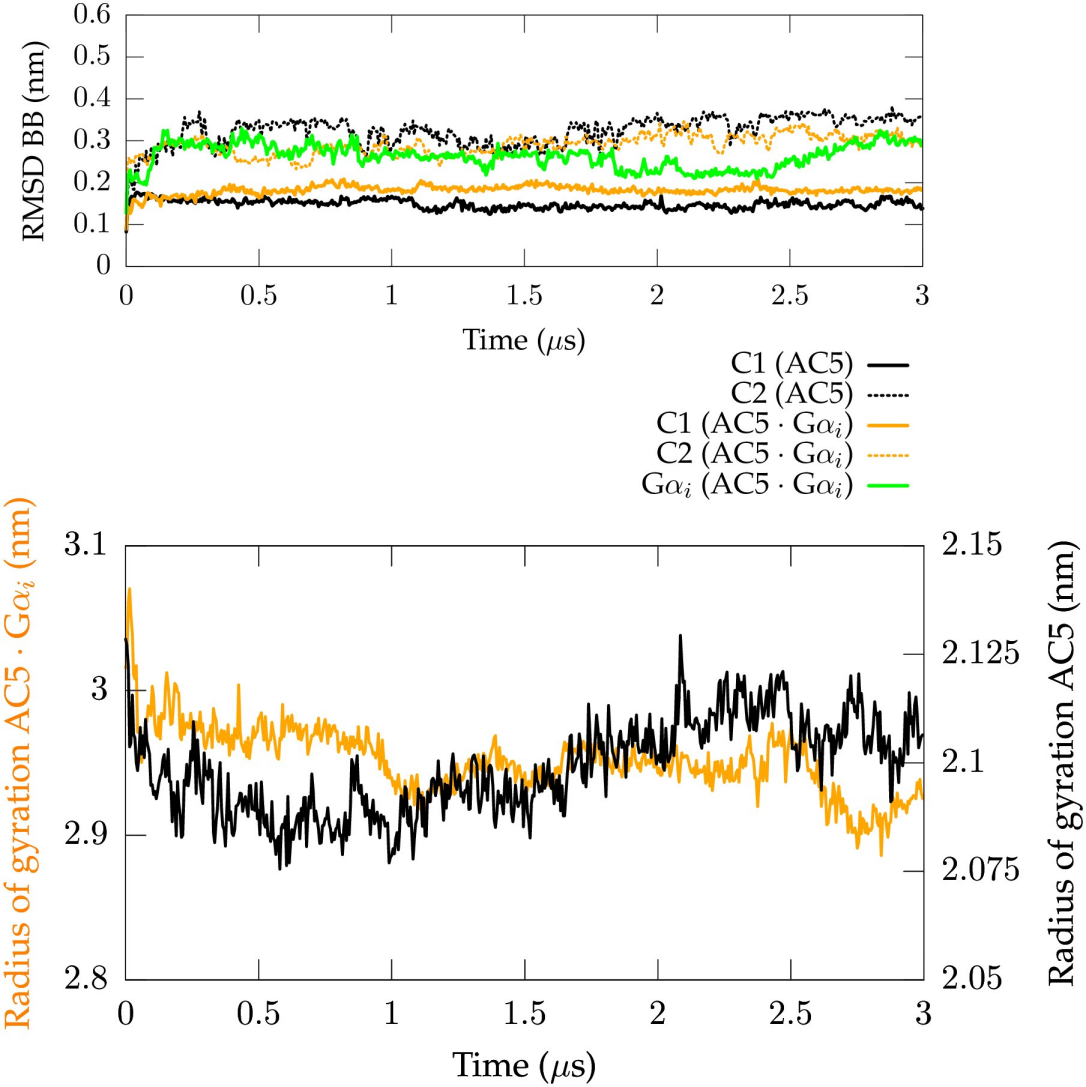
Structural stability of *holo* G*α*-free AC5 and *holo* AC5:G*α*_*i*1_ complex. Top panel: the RMSD, calculated on the protein backbone of each protein subunit with respect to the starting structure, is reported for *holo* G*α*-free AC5 and the *holo* AC5:G*α*_*i*1_ binary complex as function of time. Bottom panel: Time evolution of the radius of gyration of *holo* G*α*-free AC5 and *holo* AC5:G*α*_*i*1_ binary complex.

In order to shed light on the molecular basis of the G*α*_*i*1_ inhibition mechanism, the *holo* G*α*-free AC5 protein and the *holo* AC5:G*α*_*i*1_ complex have been simulated for 3 *μ*s. The root-mean-square deviation (RMSD) of both systems (Fig 2) shows that the subunits in each complex are stable, yet, C1 domain converges between 0.15-0.20 nm, while C2 domain stabilizes around 0.30 nm. However, the stability of the secondary structures of C1 and C2 (see S1 and S2 Figs) and the stable number of hydrogen bonds (Hbonds) in the catalytic domains (S3 Fig) all indicate subunit stability.

**Fig 3:**
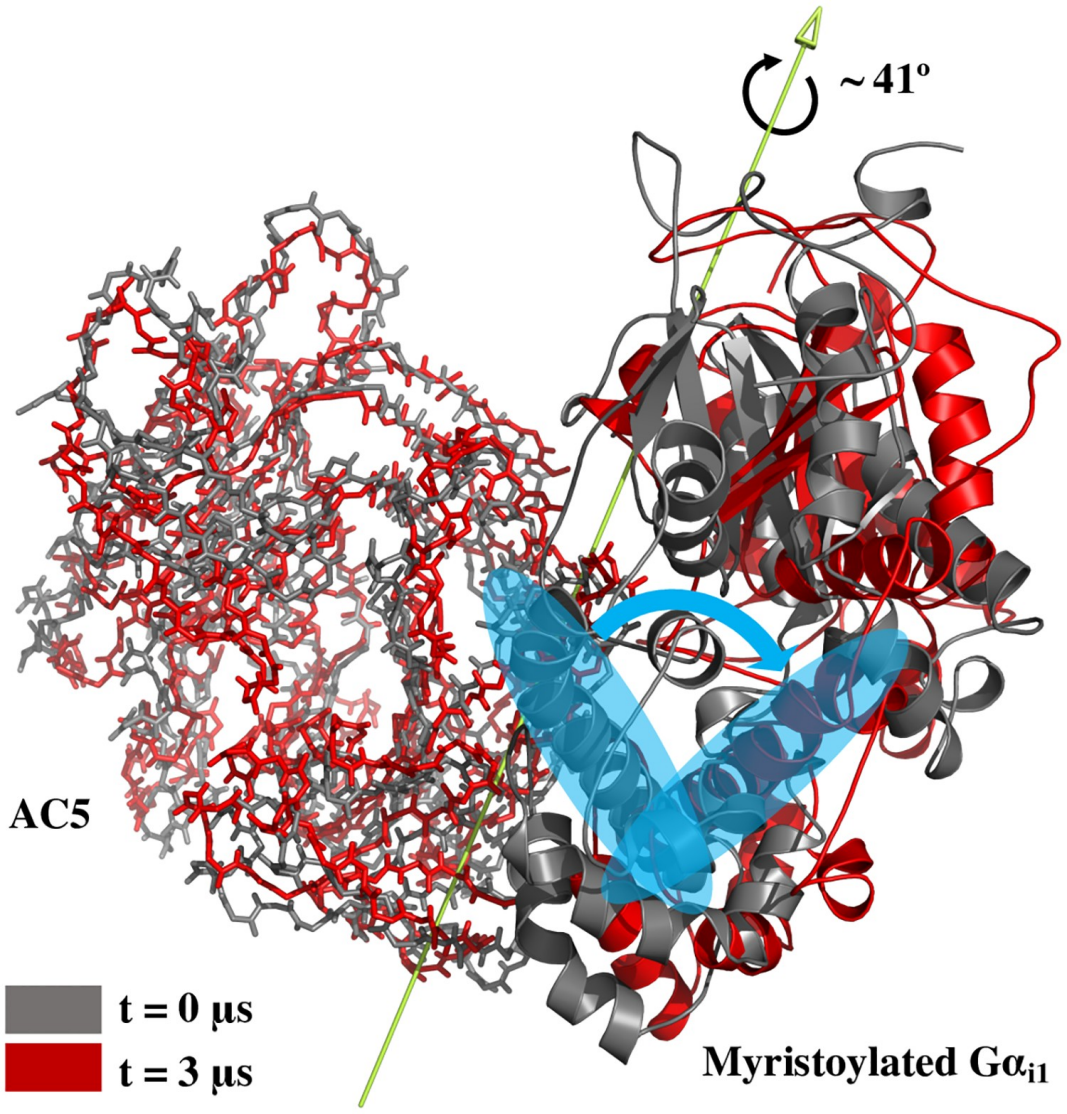
Subunits orientation in AC5:G*α*_*i*1_ complex. The protein conformation sampled in the last frame of the *holo* AC5:G*α*_*i*1_ simulation (shown in red) was compared with the first frame (shown in gray) of the same trajectory using the DynDom software. The rotational axis around which the G*α*_*i*1_ rotate of about 41°, identified by the DynDom analysis, is also shown. The rotational motion of a single helix is highlighted in order to visually identify the respective rotational mode.

Concerning the RMSD of the overall complex, *holo* G*α*-free AC5 appears to deviate significantly less from its initial structure than *holo* AC5:G*α*_*i*1_ (S4 Fig). This difference is to be expected as the initial AC5 conformation in both systems originates from an AC:G*α*_*s*_ structure in the absence of G*α*_*i*1_. Hence, in AC5:G*α*_*i*1_, AC5 is affected by G*α*_*s*_ removal as well as G*α*_*i*1_ association, inducing a larger perturbation on the AC5 protein than in the G*α*-free AC5 condition. However, the decreased radius of gyration (Rg) of the binary complex (Fig 2) implies that interdomain interactions enhance during simulations. Indeed, the number of hydrogen bonds between AC5 and G*α*_*i*1_ in the binary complex was found to increase along the simulation, which is consistent with the decrease in Rg, indicating an overall stabilization of the complex. Hence, these results suggest that the difference in overall RMSD could be due to an alteration in conformation, leading to a change in intermolecular interactions with respect to the starting structure with a possible reorientation of the system’s subunits. Such a reorientation is shown by comparing the first and the last frame of the *holo* AC5:G*α*_*i*1_ simulation using the DynDom software [44, 45] (Fig 3). This analysis identified a rotational mode defined by a rotation of ∼ 41° of the G*α*_*i*1_ protein around the rotational axis reported in Fig 3. This trend is not surprising as the initial conformation of AC5 in the binary complex originates from an AC:G*α*_*s*_ structure in the absence of G*α*_*i*1_.

The root-mean-square fluctuation (RMSF) per residue was also calculated for the C1a and C2a domains in both the G*α*-free AC5 and binary complex (S5 Fig). Concerning the C1a domain, G*α*_*i*1_ reduces the flexibility of the C-terminal part of the *α*3 helix (residues 550-560) compared to G*α*-free AC5. This change in flexibility is not surprising since this region strongly interacts with G*α*_*i*1_, resulting in reduced mobility. In the C2a domain, the *β*4′−*β*5′ strand (residues 1190-1200) is considerably less flexible in the *holo* AC5:G*α*_*i*1_ complex than in the *apo* binary and ternary AC5 complexes [27], indicating that ATP association can stabilize the mobility of the C1/C2 interface in *holo* AC5:G*α*_*i*1_ due to the positioning of the substrate.

### G*α*_*s*_ binding site

**Fig 4:**
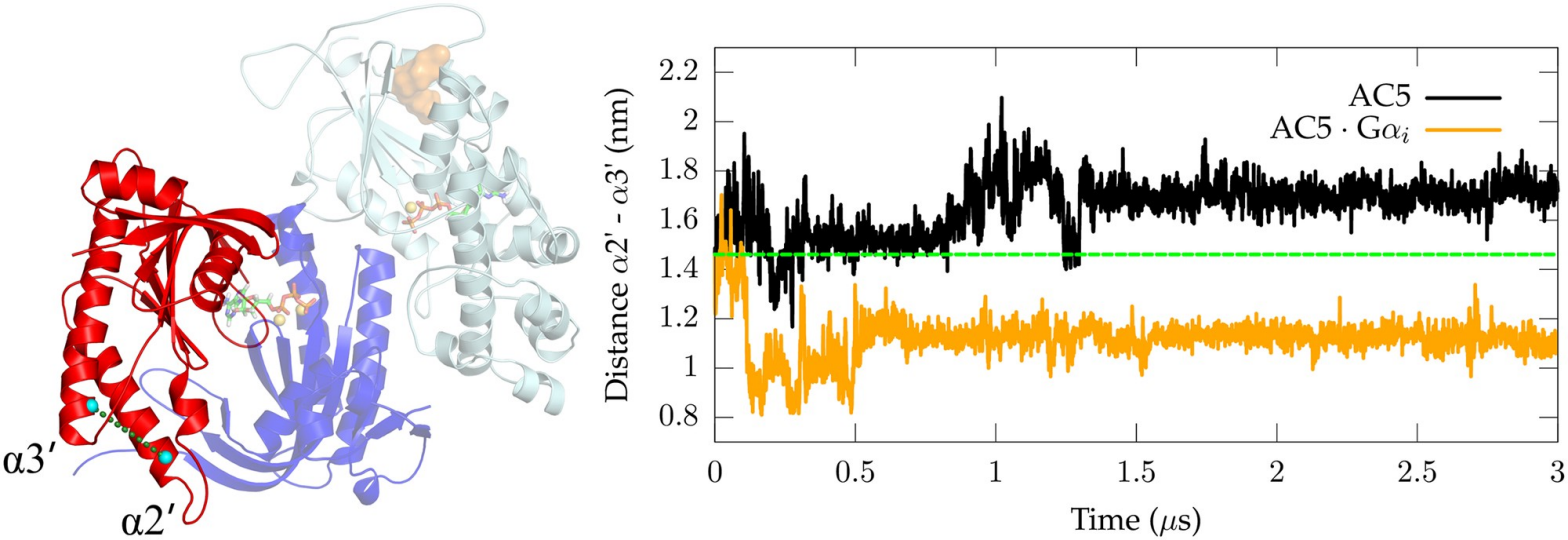
G*α*_*olf*_ binding site. On the left-hand side, the distance between the C-terminal region of *α*3’ and the N-terminal region of *α*2’ in the C2 domain of AC5 is shown as a dotted line. On the right-hand side, the time evolution of the distance between the C-terminal region of *α*3’ and the N-terminal region of *α*2’ belonging to the G*α*_*olf*_ binding site is shown for both simulated systems. The *α*2’-*α*3’ distance is calculated between the center of mass (COM) of residues 1096-1099 (*α*2’) and the COM of residues 1166-1169 (*α*3’). The green dashed line represents the starting distance between *α*2’ and *α*3’ in the two simulated systems, coincident in fair approximation to the *α*2’-*α*3’ distance in the AC:G*α*_*s*_ X-ray structure (PDB ID 1CJK).

A crucial site on C2 that could be affected by the conformational changes that occur within AC5, is the G*α*_*s*_ binding site. The effect of the presence of G*α*_*i*1_ with respect to potential G*α*_*s*_ association was investigated by comparing the conformations sampled by helix *α*2’ and *α*3’ (constituting the G*α*_*s*_ binding site) in G*α*-free AC5 and in the AC5:G*α*_*i*1_ complex (Fig 4). In the G*α*-free AC5 system, the *α*2’-*α*3’ distance (Fig 4) stabilizes around ∼1.7 nm, thus showing an increase of about 2 Å with respect to the distance in the X-ray structure of the AC:G*α*_*s*_ complex (i.e. 1.48 nm, PDB ID 1CJK) [21]. However, when G*α*_*i*1_ interacts with AC5, this distance is significantly decreased from ∼1.4 to ∼1.1 nm.

This conformational change in AC5:G*α*_*i*1_ could potentially lower the probability of G*α*_*s*_ complexation due to a reduced accessibility of the G*α*_*s*_ binding site. A decreased *α*2’-*α*3’ distance was also observed in the *apo* AC5:G*α*_*i*1_ complex [27], suggesting that such conformational change is independent from ATP association.

Moreover, the suggested reduced ability of AC5:G*α*_*i*1_ to associate with G*α*_*s*_ indirectly proposes a favourable route towards the possible formation of a *holo* ternary G*α*_*s*_:AC5:G*α*_*i*1_ complex. These results suggest that the most plausible route to form a ternary complex would be to first form *holo* AC5:G*α*_*s*_ and subsequently bind G*α*_*i*1_, which was also suggested for the *apo* form [46].

### ATP binding site

**Fig 5:**
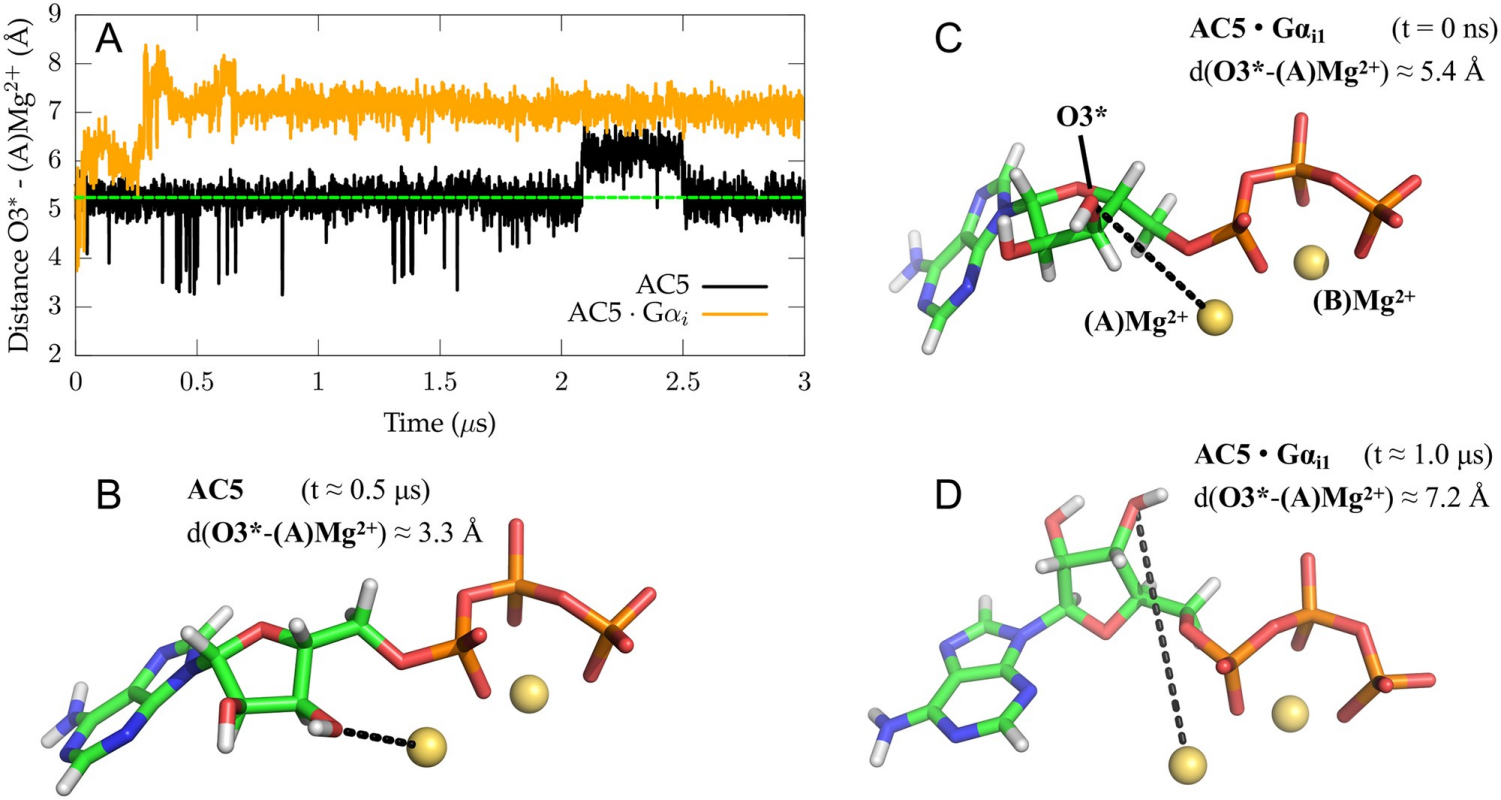
ATP sampled conformations. The distance between ATP’s ribosyl oxygen O3* and the neighbouring ${\text{Mg}}_{A}^{2+}$ is reported as function of time for both *holo* free AC5 and the AC5:G*α*_*i*1_ complex in image (A). The starting distance corresponding to the value found in the X-ray structure (PDB ID: 1CJK) is shown by a green dashed line. Snapshots of three representative conformations sampled by ATP and its neighbouring Mg^2+^ ions are shown in stick (for ATP) and ball (for Mg^2+^) representation and were extracted from the simulated trajectories of *holo* G*α*-free AC5 at 500 ns (B) and the *holo* AC5:G*α*_*i*1_ binary complex at 0 ns (C) and at 1*μ*s (D).

Although a reduced probability in G*α*_*s*_ association can negatively impact AC5’s catalytic activity as G*α*_*s*_ stimulates ATP conversion, sampling an ATP conformation that is able to convert to cAMP is imperative for AC5’s catalytic function. The first part of the conversion of ATP to cAMP consists of the deprotonation of O3* (Fig 5), which is followed by a nucleophilic attack of O3* on the phosphorous atom of the *α* phosphate, P_*α*_, producing cAMP. It is suggested that the first step of the reaction mechanism, the deprotonation of O3*, would require O3* to coordinate to the neighbouring ${\text{Mg}}_{A}^{2+}$ ion in the active site, which would lead to an ${\text{O}3*\text{-Mg}}_{A}^{2+}$ distance of ∼2.7-2.0Å [47–50]. Therefore, the distance between ATP’s O3* and ${\text{Mg}}_{A}^{2+}$ plays a crucial role and was monitored along both *holo* trajectories to study the potential catalytic activity of the conformational ensemble of ATP (Fig 5).

In the G*α*-free AC5 system, the O3*-Mg^2+^ distance was mainly found to oscillate around ∼5.4 Å (5Å<O3*-Mg^2+^<6Å is sampled in 75% of the trajectory), close to the X-ray distance of 5.25 Å (Fig 5C) [21]. To a lesser extend, a second conformation with an O3*-Mg^2+^ distance larger than 6.0 Å (O3*-Mg^2+^>6Å is sampled in 11% of the trajectory) was also sampled in G*α*-free AC5. This conformation is suggested to be inactive as the nucleophilic attack of O3* is unable to be performed at this distance (Fig 5D). Occasionally, the O3*-Mg^2+^ distance decreased to a value lower than 3.5Å in the G*α*-free AC5 simulation (O3*-Mg^2+^<3.5Å is sampled in 0.33% of the trajectory) (Fig 5B). Intriguingly, albeit poorly sampled along the simulation, this conformation could be close to the ATP structure undergoing the deprotonation step and can, therefore, be identified as the catalytically active or near-attack conformation, according to Hahn et al. [50].

ATP appears to adopt a different conformation in the AC5:G*α*_*i*1_ complex. The O3*-Mg^2+^ distance increases up to ∼8.0 Å in the first 300 ns, and equilibrates to a stable value of ∼7.2 Å for the last 2.3 *μ*s of the simulation (O3*-Mg^2+^>6Å is sampled in 96% of the trajectory), which makes the cyclization reaction unlikely to occur (Fig 5D). The absence of ${\text{O}3*\text{-Mg}}_{A}^{2+}$ coordination seems to indicate an inhibited state of ATP in the binary complex as a near-attack conformation of ATP [21] cannot be sampled. Additionally, the main inactive conformation sampled by ATP in AC5:G*α*_*i*1_ adopts a structure incapable of sampling other states, whereas in the G*α*-free AC5 system, an equilibrium between the different ATP conformations is already present on the microsecond timescale. In order to check the reproducibility of the observed conformational change of ATP in AC5:G*α*_*i*1_, a second MD simulation of the *holo* AC5:G*α*_*i*1_ complex was performed using different initial velocities. This simulation of ∼0.7 *μ*s confirms the presence of an inactive conformation of ATP in AC5:G*α*_*i*1_ since the same trend in conformational change was observed for the nucleotide as in the first AC5:G*α*_*i*1_ simulation (S6 Fig).

**Fig 6:**
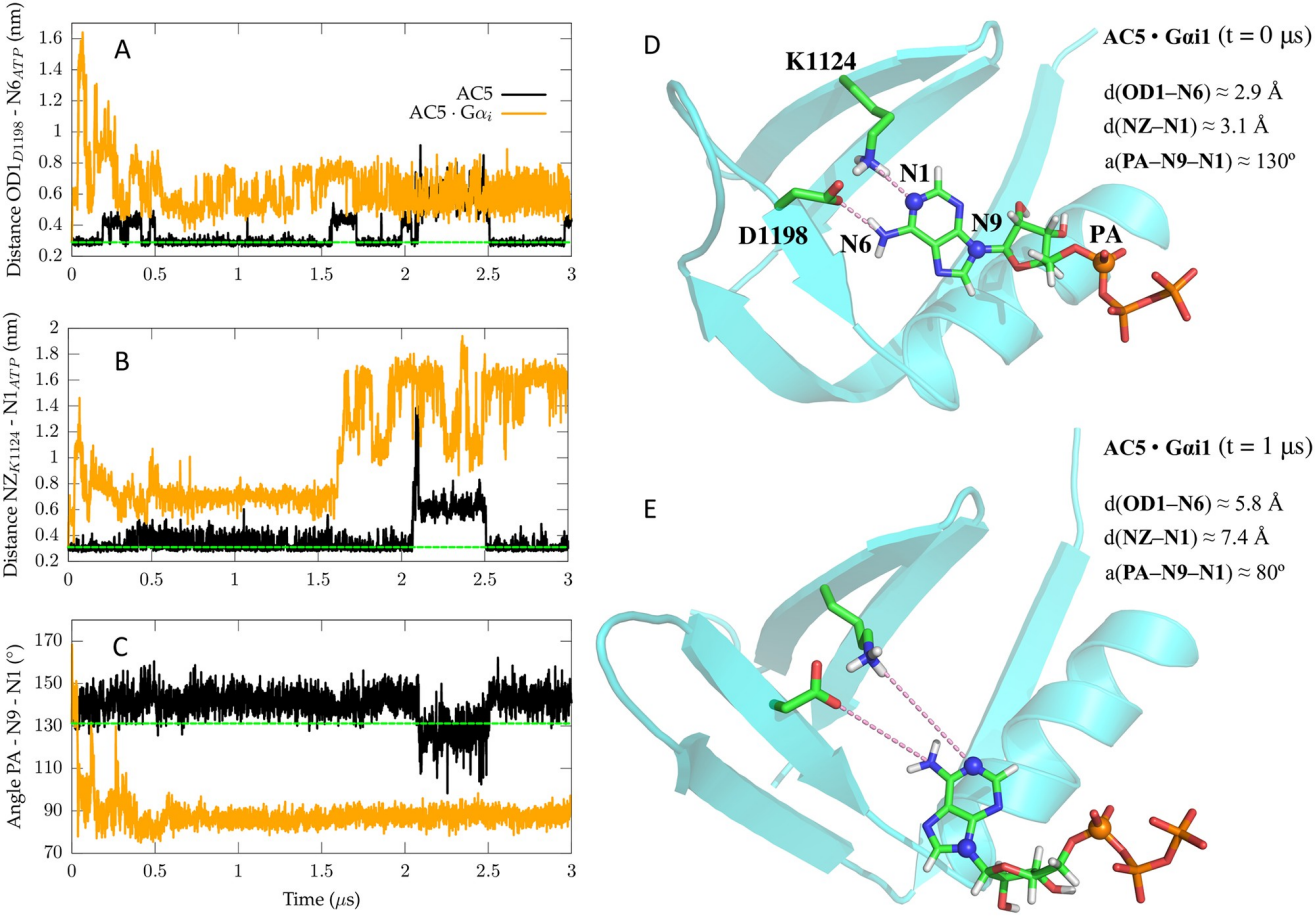
ATP’s adenine orientation in the active site of AC5:G*α*_*i*1_ and G*α*-free AC5. Distances between the adenine moiety of ATP and D1198 (A), D1198(OD1)-ATP(N6), as well as K1124 (B), K1124(NZ)-ATP(N1) are shown. The position of these residues with respect to ATP is depicted in images (D) and (E). The dashed green line indicates the value for each initial distance in the AC5:G*α*_*i*1_ and G*α*-free AC5 systems. (C) is a representation of an angle between ATP’s P_*α*_ and two nitrogens in the adenine group, ATP(PA-N9-N1), which are highlighted in image (D). The dashed green line indicates the value of the initial angle in the AC5:G*α*_*i*1_ and G*α*-free AC5 systems. (D) and (E) show the interactions between ATP and two residues, K1124 and D1198, at the beginning and at the end of the simulation of the AC5:G*α*_*i*1_ system. ATP, K1124 and D1198 are represented in licorice (carbon in green, oxygen in red, nitrogen in blue, phosphor in orange and hydrogen in white) and the C2 domain is highlighted in cyan in cartoon representation. Distances between ATP and the two residues are shown in dashed pink lines with the corresponding values in Ångstrom displayed in the right corner. The atoms used in the calculation of the angle plotted in (C) are depicted via blue and orange spheres, which are labeled in image (D).

The inactive ATP conformation sampled in AC5:G*α*_*i*1_ could be the result of conformational changes at the C1/C2 interface, perturbing interactions between ATP and AC5’s active site. When comparing AC5:G*α*_*i*1_ and G*α*-free AC5, a crucial difference seems to be a change in ATP’s interactions with the C2 domain around its adenosine group which takes place in the first 200 ns of the simulation. Whereas in the G*α*-free AC5 system the interactions of the adenine moiety and the active site are stable (Fig 6), an irreversible loss of a hydrogen bond can be observed in the AC5:G*α*_*i*1_ system between adenine and K1124, connected to a second hydrogen bond with D1198 (Fig 6, S7 and S8 Figs). The loss of the K1124-ATP and D1198-ATP hydrogen bonds appears to destabilize the position of the adenine ring in the active site, which reorients shortly after (Fig 6, S8 Fig). The conformational change of the adenine ring in turn seems to impact the interaction of ATP with ${\text{Mg}}_{A}^{2+}$ as it appears to coincide with the increase in distance between O3* and ${\text{Mg}}_{A}^{2+}$, leading to the suggested inactive state of ATP (Figs 5 and 6). This mechanism was also observed in the second MD simulation of AC5:G*α*_*i*1_ (S6–S8 Figs).

Both K1124 and D1198 are conserved across species (rat, dog, human, rabbit and mouse) and are also conserved between all membrane-bound isoforms of AC (AC1-9 in mouse). The conservation of these two residues in the active site could indicate that both residues indeed play important roles in maintaining the catalytic function of adenylyl cyclase. The performed simulations suggest that K1124 and D1198 are crucial for stabilizing the conformation of ATP in the active site and when these interactions are lost, an inactive ATP state is sampled.

Hence, these results suggest the conformational change of the ATP substrate into an inactive state is promoted by G*α*_*i*1_:AC5 complex formation. Although G*α*_*i*1_ association appears to inhibit the catalytic function of AC5 destabilizing the active conformation of ATP, it cannot be excluded that G*α*_*i*1_ could also affect AC5’s activity by impairing its substrate binding affinity. However, it was experimentally determined by Dessauer and coworkers [51] that Km does not significantly change upon G*α*_*i*_ or G*α*_*s*_ binding. Therefore, the G*α*_*i*1_-induced conformational change of ATP in AC5’s active site seems to be the main effect of G*α*_*i*1_’s inhibition mechanism on AC5.

## Conclusions

Here, the structural and dynamical properties of the AC5:G*α*_*i*1_ complex have been characterized in order to understand the inhibition mechanism triggered by G*α*_*i*1_ association. The conformation adopted by the ATP substrate in the binary complex significantly reduces the probability of an S_*N*_2 reaction between O3* and P*α*, potentially preventing the formation of cAMP. These findings demonstrate that although ATP is able to interact with the C1/C2 interface in both AC5:G*α*_*i*1_ and G*α*-free AC5 systems, the catalytic activity of AC5 seems to be impaired by G*α*_*i*1_ association, affecting the conformation of the substrate. Additionally, these results show that, as already observed in the *apo* form, also in *holo* AC5, G*α*_*i*1_ association induces a conformational change in the G*α*_*s*_ binding region on the AC5 C2 domain, thus resulting in a closure of this binding site. Based on this observation it is suggested that G*α*_*i*1_ association prevents ternary complex formation via this route. This potential pathway, found in previous studies of the *apo* form, is also reported here for the *holo* form and confirms the existence of a potentially favourable route towards ternary complex formation from the AC5:G*α*_*s*_ complex.

## Supporting information

S1 Fig Secondary structures *holo* G*α*-free AC5. Time evolution of the secondary structures of AC5 as obtained by DSSP analysis on the trajectory of *holo* G*α*-free AC5.
(TIF)

S2 Fig Secondary structures *holo* AC5:G*α*_*i*1_. Time evolution of the secondary structures of AC5 (top panel) and G*α*_*i*1_ (bottom panel) as obtained by DSSP analysis on the trajectory of the *holo* AC5:G*α*_*i*1_ binary complex.
(TIF)

S3 Fig Hydrogen bonds analysis. Top panel: Number of hydrogen bonds (H-bond) present in the AC5 protein calculated on the *holo* G*α*-free AC5 trajectory (black line), and in the AC5 protein (blue line) and G*α*_*i*1_ subunit (green line) of the *holo* AC5:G*α*_*i*1_ trajectory as function of time. Bottom panel: Time evolution of the number of hydrogen bonds formed between AC5 and G*α*_*i*1_ along the simulated trajectory of the *holo* AC5:G*α*_*i*1_ binary complex.
(TIF)

S4 Fig RMSDs of *Holo* AC5 complexes. Root-mean-square deviation (RMSD), calculated on the protein backbone with respect to the starting structure of each protein subunit or the complete complex, is reported for *holo* G*α*-free AC5 and the *holo* AC5:G*α*_*i*1_ binary complex as function of time.
(TIF)

S5 Fig Structural fluctuations. Root-mean-square fluctuations (RMSFs) of the C1 (top panel) and C2 (bottom panel) domains calculated on the protein backbone for both simulated systems, *holo* G*α*-free and AC5:G*α*_*i*1_, excluding the first microsecond of simulation and block-averaged over 100 ns segments. RMSFs of the *apo* AC5:G*α*_*i*1_ system are also reported in cyan.
(TIF)

S6 FigATP sampled conformations.The distance between ATP’s ribosyl oxygen O3* and the neighbouring ${\text{Mg}}_{A}^{2+}$ is reported as function of time in the first *μ*s of simulation of *holo* free AC5 and *holo* AC5:G*α*_*i*1_ complex. In the case of *holo* AC5:G*α*_*i*1_ complex the same analysis was repeated on a second independent trajectory (blue line). The starting distance corresponding to the value found in the X-ray structure (PDB ID: 1CJK) is shown by a green dashed line.(TIF)

S7 Fig ATP’s adenine orientation in the active site of AC5:G*α*_*i*1_ and G*α*-free AC5. Distances between the adenine moiety of ATP and D1198 (A), D1198(OD1)-ATP(N6), as well as K1124 (B), K1124(NZ)-ATP(N1), are shown for the two simulations performed for the AC5:G*α*_*i*1_ system (blue and orange) and the G*α*-free AC5 trajectory (black). The dashed green line indicates the value for each initial distance in the AC5:G*α*_*i*1_ and G*α*-free AC5 systems. (C) is a representation of an angle between ATP’s P_*α*_ and two nitrogens in the adenine group, ATP(PA-N9-N1), which are highlighted in Fig 6. This plot depicts the two simulations performed for the AC5:G*α*_*i*1_ system (blue and orange) and the G*α*-free AC5 trajectory (black). The dashed green line indicates the value of the initial angle in the AC5:G*α*_*i*1_ and G*α*-free AC5 systems.
(TIF)

S8 FigConformational changes of ATP in the active site of AC5:G*α*_*i*1_.Distances between the adenine moiety of ATP and D1198 (A), D1198(OD1)-ATP(N6), as well as K1124 (B), K1124(NZ)-ATP(N1), are shown for the AC5:G*α*_*i*1_ system for the first 200 ns of the trajectory. The dashed green line indicates the value for each initial distance in the AC5:G*α*_*i*1_ system. The complete trajectory is represented in Fig 6. (C) Angle between ATP’s P_*α*_ and two nitrogens in the adenine group, ATP(PA-N9-N1), is shown of the first 200 ns of the AC5:G*α*_*i*1_ simulation reported in Fig 6. The dashed green line indicates the value of the initial angle in the AC5:G*α*_*i*1_ and G*α*-free AC5 systems. (D) Distance between ATP’s ribosyl oxygen O3* and the neighbouring ${\text{Mg}}_{A}^{2+}$ reported as function of time for the first 200 ns of simulation in the AC5:G*α*_*i*1_ system. The complete trajectory is represented in Fig 6. The starting distance corresponding to the value found in the X-ray structure (PDB ID: 1CJK) is shown by a green dashed line. (E,F,G,H) represent the respective values reported in (A,B,C,D) obtained in the second run of the AC5:G*α*_*i*1_ system, which was simulated for ∼0.7*μ*s in total (S7 Fig).(TIF)

S1 File (ZIP)

## References

[1] Tang WJ, Gilman AG. Adenylyl cyclases. Cell. 1992;70:869–872. 10.1016/0092-8674(92)90236-6 1525824

[2] Sunahara RK, Taussig R. Isoforms of mammalian adenylyl cyclase: multiplicities of signaling. Mol Inter. 2002;2:168–184. 10.1124/mi.2.3.168 14993377

[3] Sunahara RK, Dessauer CW, Gilman AG. Complexity and diversity of mammalian adenylyl cyclases. Annu Rev Pharmacol Toxicol. 1996;36:461–480. 10.1146/annurev.pa.36.040196.002333 8725398

[4] Gray P, Scott J, Catterall W. Regulation of ion channels by cAMP-dependent protein kinase and A-kinase anchoring proteins. Curr Opin Neurobiol. 1998;8:330–334. 10.1016/S0959-4388(98)80057-3 9687361

[5] Pedarzani P, Storm JF. Protein kinase A-independent modulation of ion channels in the brain by cyclic AMP. Proc Natl Acad Sci USA. 1995;92:11716–11720. 10.1073/pnas.92.25.11716 8524835 PMC40473

[6] Taylor SS, Zhang P, Steichen JM, Keshwani MM, Kornev AP. PKA: lessons learned after twenty years. Biochim Biophys Acta. 2013;1834:1271–1278. 10.1016/j.bbapap.2013.03.007 23535202 PMC3763834

[7] Delmeire D, Flamez D, Hinke SA, Cali JJ, Pipeleers D, Schuit F. Type VIII adenylyl cyclase in rat beta cells: coincidence signal detector/generator for glucose and GLP-1. Diabetologia. 2003;46:1383–1393. 10.1007/s00125-003-1203-8 13680124

[8] McVey M, Hill J, Howlett A, Klein C. Adenylyl cyclase, a coincidence detector for nitric oxide. J Biol Chem. 1999;274:18887–18892. 10383385 10.1074/jbc.274.27.18887

[9] Nair AG, Gutierrez-Arenas O, Eriksson O, Vincent P, Kotaleski JH. Sensing Positive versus Negative Reward Signals through Adenylyl Cyclase-Coupled GPCRs in Direct and Indirect Pathway Striatal Medium Spiny Neurons. J Neurosc. 2015;35:14017–14030. 10.1523/JNEUROSCI.0730-15.2015 26468202 PMC4604235

[10] Anholt RRH. Signal integration in the nervous system: adenylate cyclases as molecular coincidence detectors. Trends in Neurosciences. 1994;17:37–41. 10.1016/0166-2236(94)90033-7 7511849

[11] Bourne HR, Nicoll R. Molecular machines integrate coincident synaptic signals. Cell. 1993;72:65–75. 10.1016/S0092-8674(05)80029-7 8094038

[12] Impey S, Wayman G, Wu Z, Storm DR. Type I Adenylyl Cyclase Functions as a Coincidence Detector for Control of Cyclic AMP Response Element-Mediated Transcription: Synergistic Regulation of Transcription by Ca^2+^ and Isoproterenol. Mol Cell Biol. 1994;14:8272–8281. 10.1128/mcb.14.12.8272 7969163 PMC359366

[13] Lustig KD, Conklin BR, Herzmark P, Taussig R, Bourne HR. Type II adenylylcyclase integrates coincident signals from Gs, Gi, and Gq. J Biol Chem. 1993;268:13900–13905. 10.1016/S0021-9258(19)85187-6 8390980

[14] Mons N, Guillou JL, Jaffard R. The role of Ca2+/calmodulin-stimulable adenylyl cyclases as molecular coincidence detectors in memory formation. Cell Mol Life Sci. 1999;55:525–533. 10.1007/s000180050311 10357223 PMC11147090

[15] Glatt CE, Snyder SH. Cloning and expression of an adenylyl cyclase localized to the corpus striatum. Nature. 1993;361:536–538. 10.1038/361536a0 8429907

[16] Mons M, Cooper DMF. Selective expression of one Ca^2+^-inhibitable adenylyl cyclase in dopaminergically innervated rat brain regions. Molecular Brain Research. 1994;22:236–244. 10.1016/0169-328X(94)90052-3 8015383

[17] Cabrera-Vera TM, Vanhauwe J, Thomas TO, Medkova M, Preininger A, Mazzoni MR, et al Insights into G protein structure, function, and regulation. Endocrine Rev. 2003;24:765–781. 10.1210/er.2000-0026 14671004

[18] Dessauer CW, Tesmer JJG, Sprang SR, Gilman AG. Identification of a Gi*α* binding site on type V adenylyl cyclase. J Biol Chem. 1998;273:25831–25839. 9748257 10.1074/jbc.273.40.25831

[19] Taussig R, Iniguez-Lluhi JA, Gilman AG. Inhibition of adenylyl cyclase by Gi alpha. Science. 1993;261:218–221. 10.1126/science.8327893 8327893

[20] Taussig R, Tang WJ, Hepler JR, Gilman AG. Distinct patterns of bidirectional regulation of mammalian adenylyl cyclases. J Biol Chem. 1994;269:6093–6100. 10.1016/S0021-9258(17)37574-9 8119955

[21] Tesmer JJG, Sunahara RK, Gilman AG, Sprang SR. Crystal structure of the catalytic domains of adenylyl cyclase in a complex with Gs*α*· GTP*γ*S. Science. 1997;278:1907–1916. 10.1126/science.278.5345.1907 9417641

[22] Tesmer JJG, Sunahara RK, Johnson RA, Gosselin G, Gilman AG, Sprang SR. Two-metal-ion catalysis in adenylyl cyclase. Science. 1999;285:756–760. 10.1126/science.285.5428.756 10427002

[23] Qi C, Sorrentino S, Medalia O, Korkhov VM. The structure of a membrane adenylyl cyclase bound to an activated stimulatory G protein. Science. 2019;364:389–394. 10.1126/science.aav0778 31023924

[24] Whisnant RE, Gilman AG, Dessauer CW. Interaction of the two cytosolic domains of mammalian adenylyl cyclase. Proc Nat Ac Sci USA. 1996;93:6621–6625. 10.1073/pnas.93.13.6621 8692867 PMC39075

[25] Preininger AM, Van Eps N, Yu NJ, Medkova M, Hubbell WL, Hamm HE. The myristoylated amino terminus of G*α*i1 plays a critical role in the structure and function of G*α*i1 subunits in solution. Biochemistry. 2003;42:7931–7941. 10.1021/bi0345438 12834345

[26] van Keulen SC, Rothlisberger U. Exploring the Inhibition Mechanism of Adenylyl Cyclase Type 5 by N-terminal Myristoylated G*α*_*i*1_:GTP. PLoS Comp Biol. 2017;13:e1005673 10.1371/journal.pcbi.1005673 PMC5608429 28892485

[27] van Keulen SC, Narzi D, Rothlisberger U. Association of Both Inhibitory and Stimulatory G*α* Subunits Implies Adenylyl Cyclase 5 Deactivation. Biochemistry. 2019;58:4317–4324. 10.1021/acs.biochem.9b00662 31525953

[28] Frezza E, Martin J, Lavery R. A molecular dynamics study of adenylyl cyclase: The impact of ATP and G-protein binding. PLoS ONE. 2018;13:e0196207 10.1371/journal.pone.0196207 29694437 PMC5918993

[29] Van Keulen SC, Rothlisberger U. Effect of N-Terminal Myristoylation on the Active Conformation of G*α* i 1–GTP. Biochemistry. 2016;56:271–280. 10.1021/acs.biochem.6b00388 27936598

[30] De Vries SJ, van Dijk M, Bonvin AMJJ. The HADDOCK web server for data-driven biomolecular docking. Nature Protocols. 2010;5:883–897. 10.1038/nprot.2010.32 20431534

[31] Hornak V, Abel R, Okur A, Strockbine B, Roitberg A, Simmerling C. Comparison of multiple Amber force fields and development of improved protein backbone parameters. Prot Struct Funct Bioinf. 2006;65:712–725. 10.1002/prot.21123 16981200 PMC4805110

[32] Jorgensen WL, Chandrasekhar J, Madura JD, Impey RW, Klein ML. Comparison of simple potential functions for simulating liquid water. J Chem Phys. 1983;79:926–935. 10.1063/1.445869

[33] Bekker H, Berendsen HJC, Dijkstra EJ, Achterop S, Van Drunen R, Van der Spoel D, et al Gromacs: A parallel computer for molecular dynamics simulations. Physics Computing. 1993;92:252–256.

[34] Hess B, Kutzner C, Van Der Spoel D, Lindahl E. GROMACS 4: algorithms for highly efficient, load-balanced, and scalable molecular simulation. J Chem Theory Comp. 2008;4:435–447. 10.1021/ct700301q 26620784

[35] Joung IS, Cheatham TE III. Determination of alkali and halide monovalent ion parameters for use in explicitly solvated biomolecular simulations. J Phys Chem B. 2008;112:9020–9041. 10.1021/jp8001614 18593145 PMC2652252

[36] Meagher KL, Redman LT, Carlson HA. Development of polyphosphate parameters for use with the AMBER force field. J Comput Chem. 2003;24:1016–1025. 10.1002/jcc.10262 12759902

[37] Allnér O, Nilsson L, Villa A. Magnesium ion–water coordination and exchange in biomolecular simulations. J Chem Theory Comp. 2012;8:1493–1502. 10.1021/ct3000734 26596759

[38] Nosé S. A unified formulation of the constant temperature molecular dynamics methods. J Chem Phys. 1984;81:511–519. 10.1063/1.447334

[39] Hoover WG. Canonical dynamics: Equilibrium phase-space distribution. Phys Rev A. 1985;31:1695–1697. 10.1103/PhysRevA.31.1695 9895674

[40] Parrinello M, Rahman A. Polymorphic transitions in single crystals: A new molecular dynamics method. J Appl Phys. 1981;52:7182–7190. 10.1063/1.328693

[41] Darden TA, York DM, Pedersen LG. Particle mesh Ewald: “An *N*log(*N*) method for Ewald sums in large systems”. J Chem Phys. 1993;98:10089–10092. 10.1063/1.464397

[42] Hess B. P-LINCS: A parallel linear constraint solver for molecular simulation. J Chem Theory Comp. 2008;4:116–122. 10.1021/ct700200b 26619985

[43] Schrödinger, LLC. The PyMOL Molecular Graphics System, Version 1.8; 2015.

[44] Hayward S, Berendsen H. Systematic analysis of domain motions in proteins from conformational change: new results on citrate synthase and T4 lysozyme. Proteins. 1998;30:144–154. 10.1002/(SICI)1097-0134(19980201)30:2<144::AID-PROT4>3.0.CO;2-N 9489922

[45] Hayward S, Lee R. Improvements in the analysis of domain motions in proteins from conformational change: DynDom version 1.50. J Mol Graph Model. 2002;21:181–183. 10.1016/S1093-3263(02)00140-7 12463636

[46] Bruce N, Narzi D, Trpevski D, van Keulen SC, Nair A, Vidossich P, et al Regulation of adenylyl cyclase 5 in striatal neurons confers the ability to detect coincident neuromodulatory signals. PLoS Comp Biol. 2019;15:e1007382 10.1371/journal.pcbi.1007382 31665146 PMC6821081

[47] Steitz TA. DNA-and RNA-dependent DNA polymerases. Curr Opin Str Biol. 1993;3:31–38. 10.1016/0959-440X(93)90198-T

[48] Tesmer JJG, Sprang SR. The structure, catalytic mechanism and regulation of adenylyl cyclase. Curr Opin Str Biol. 1998;8:713–719. 10.1016/S0959-440X(98)80090-0 9914249

[49] Nakamura T, Zhao Y, Yamagata Y, Hua YJ, Yang W. Watching DNA polymerase *η* make a phosphodiester bond. Nature. 2012;487:196–201. 10.1038/nature11181 22785315 PMC3397672

[50] Hahn DK, Tusell JR, Sprang SR, Chu X. Catalytic Mechanism of Mammalian Adenylyl Cyclase: A Computational Investigation. Biochemistry. 2015;54:6252–6262. 10.1021/acs.biochem.5b00655 26393535 PMC5156327

[51] Chen-Goodspeed M, Lukan AN, Dessauer CW. Modeling of G*α*_*s*_ and G*α*_*i*_ Regulation of Human Type V and VI Adenylyl Cyclase. J Biol Chem. 2005;280:1808–1816. 15545274 10.1074/jbc.M409172200

