# Supplementary figures and images for: Gαi1 inhibition mechanism of ATP-bound adenylyl cyclase type 5
Source: PLoS One. 2021 Jan 25;16(1):e0245197. doi: 10.1371/journal.pone.0245197 (PMC7833170; doi:10.1371/journal.pone.0245197)

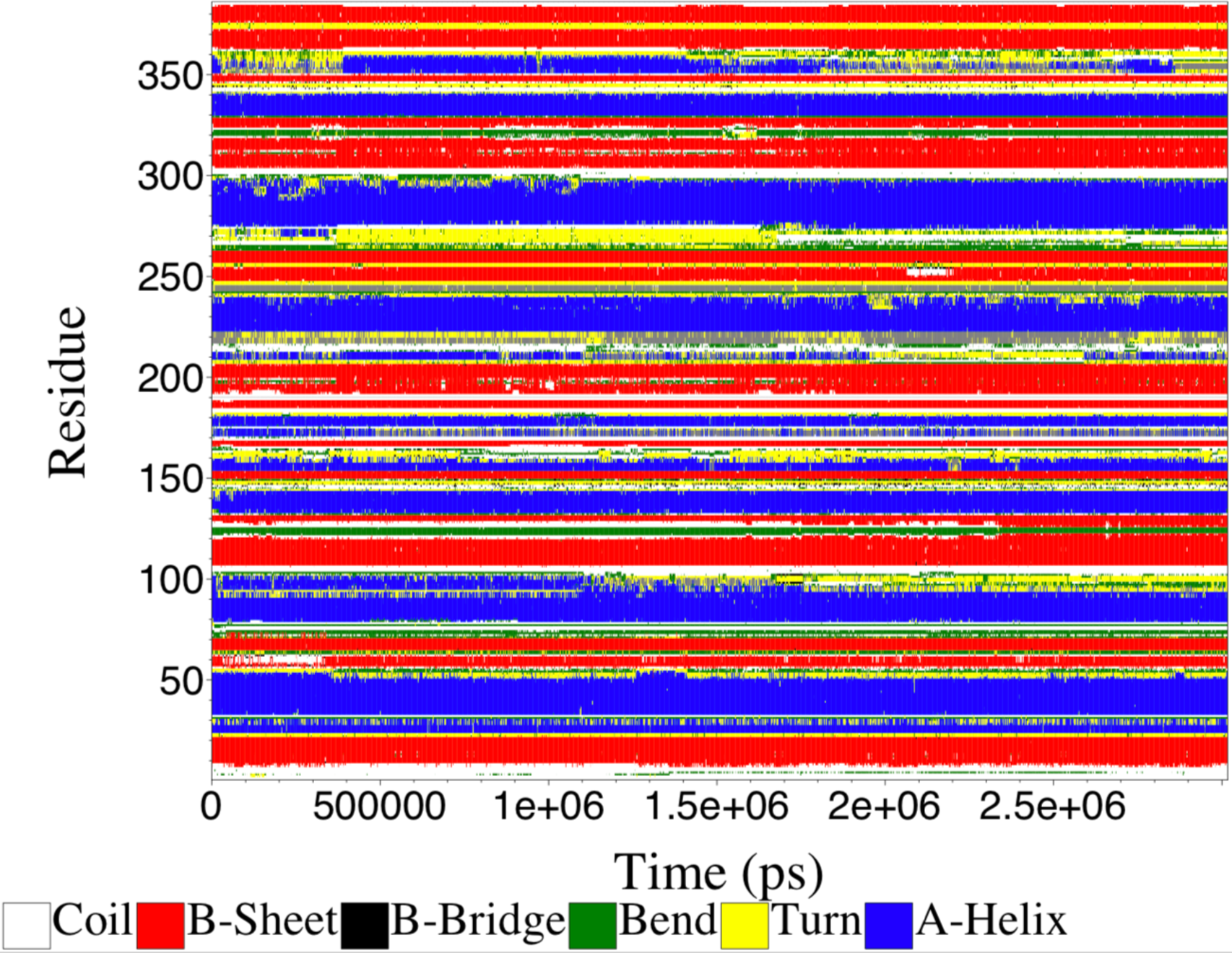

Supplement: S1 Fig — Time evolution of the secondary structures of AC5 as obtained by DSSP analysis on the trajectory of holo Gα-free AC5. (TIF) [file pone.0245197.s001.tif]

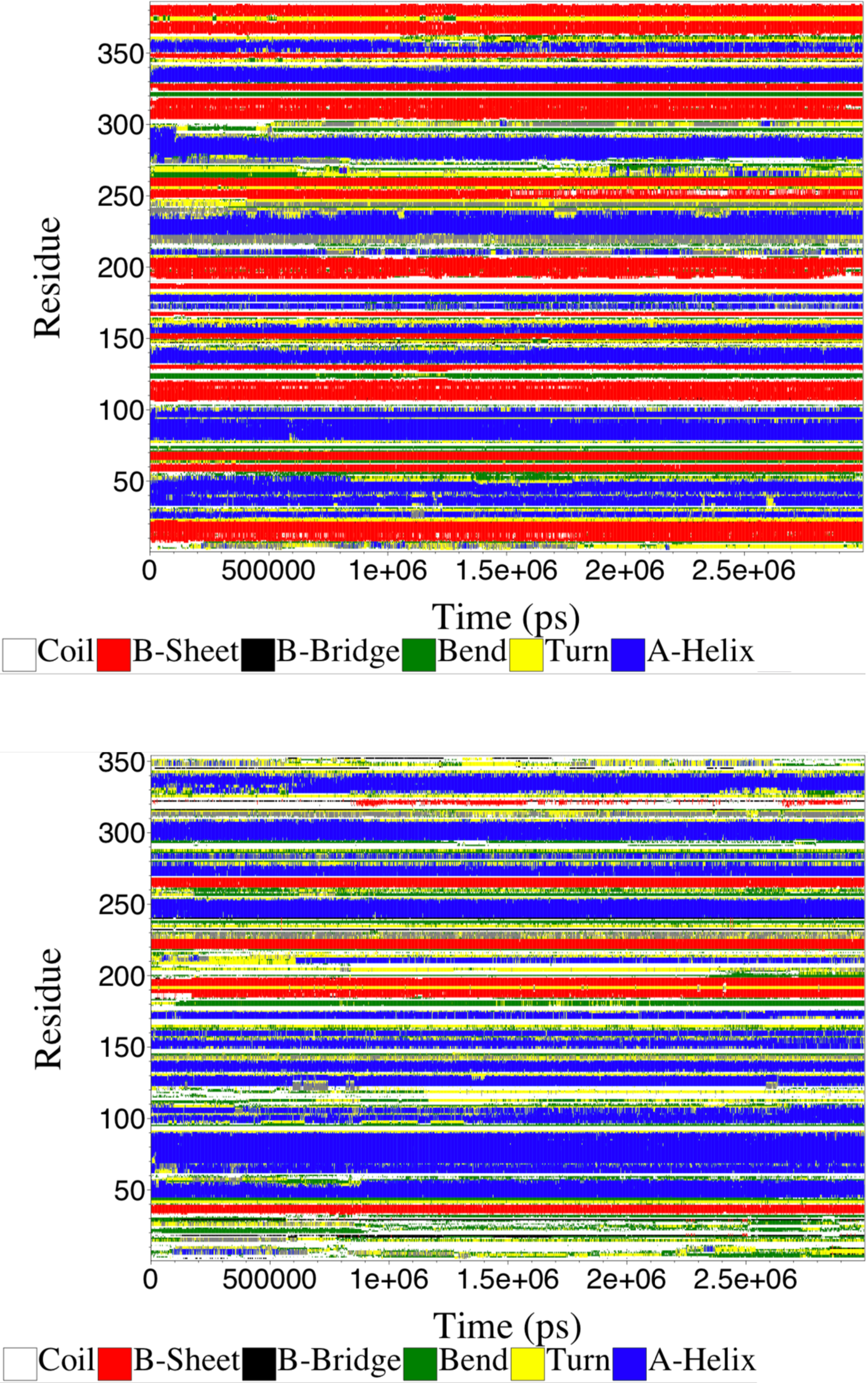

Supplement: S2 Fig — Time evolution of the secondary structures of AC5 (top panel) and Gαi1 (bottom panel) as obtained by DSSP analysis on the trajectory of the holo AC5:Gαi1 binary complex. (TIF) [file pone.0245197.s002.tif]

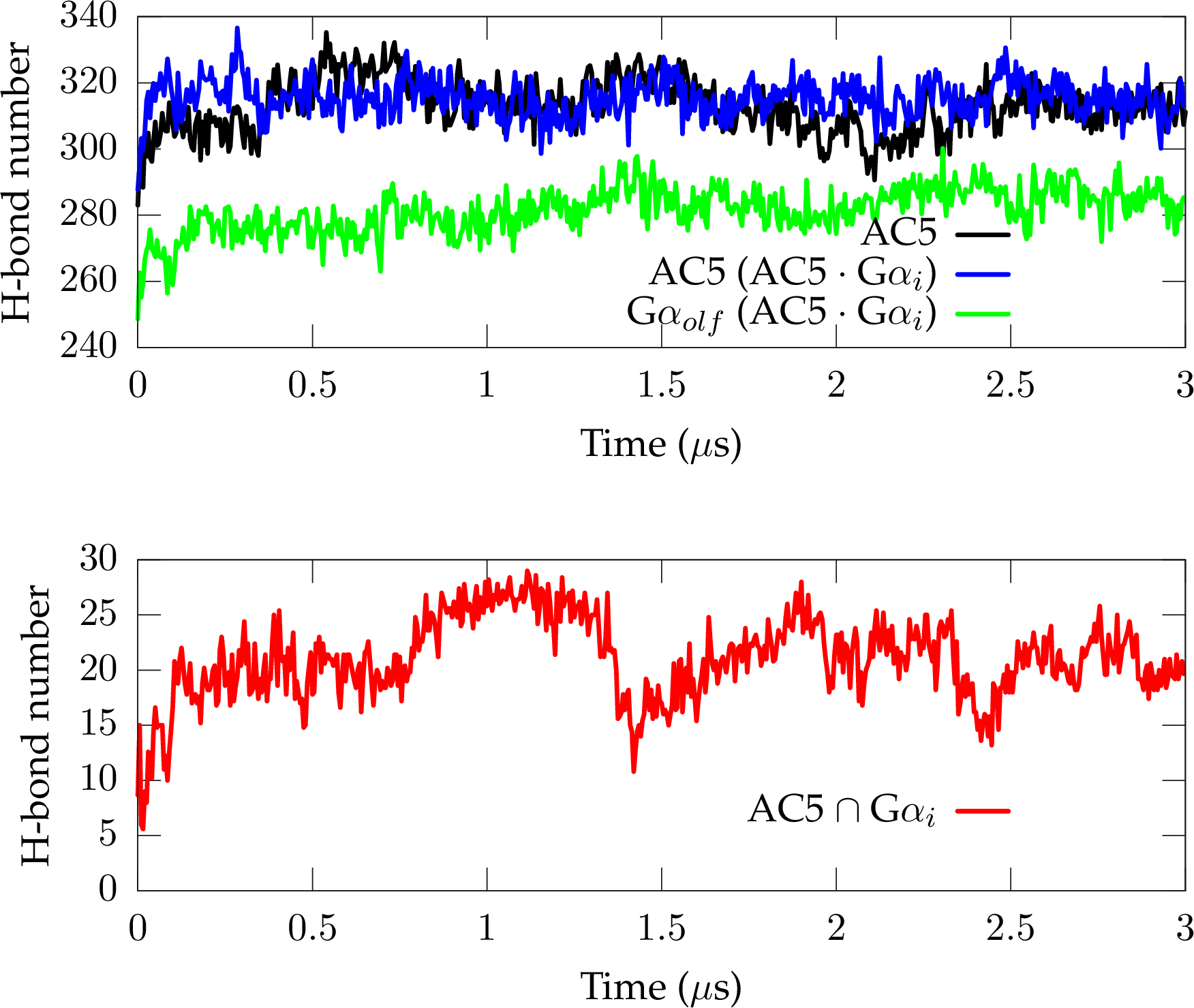

Supplement: S3 Fig — Top panel: Number of hydrogen bonds (H-bond) present in the AC5 protein calculated on the holo Gα-free AC5 trajectory (black line), and in the AC5 protein (blue line) and Gαi1 subunit (green line) of the holo AC5:Gαi1 trajectory as function of time. Bottom panel: Time evolution of the number of hydrogen bonds formed between AC5 and Gαi1 along the simulated trajectory of the holo AC5:Gαi1 binary complex. (TIF) [file pone.0245197.s003.tif]

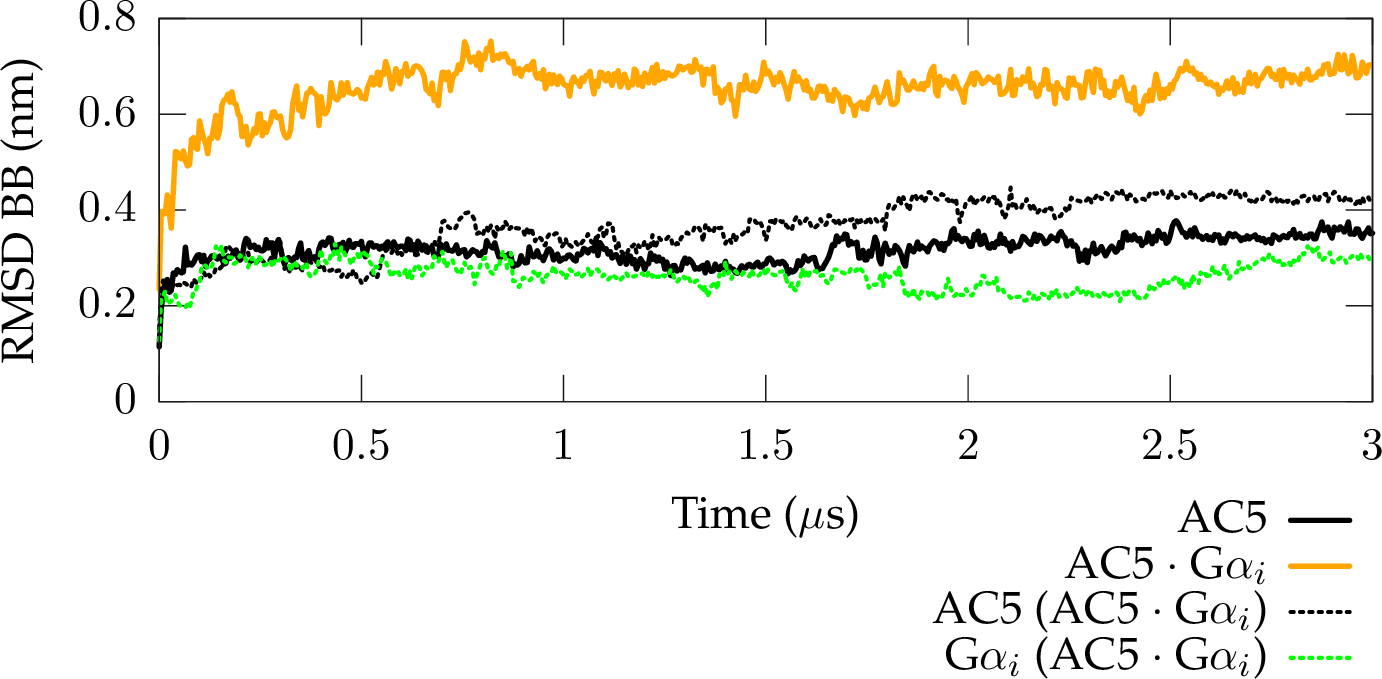

Supplement: S4 Fig — Root-mean-square deviation (RMSD), calculated on the protein backbone with respect to the starting structure of each protein subunit or the complete complex, is reported for holo Gα-free AC5 and the holo AC5:Gαi1 binary complex as function of time. (TIF) [file pone.0245197.s004.tif]

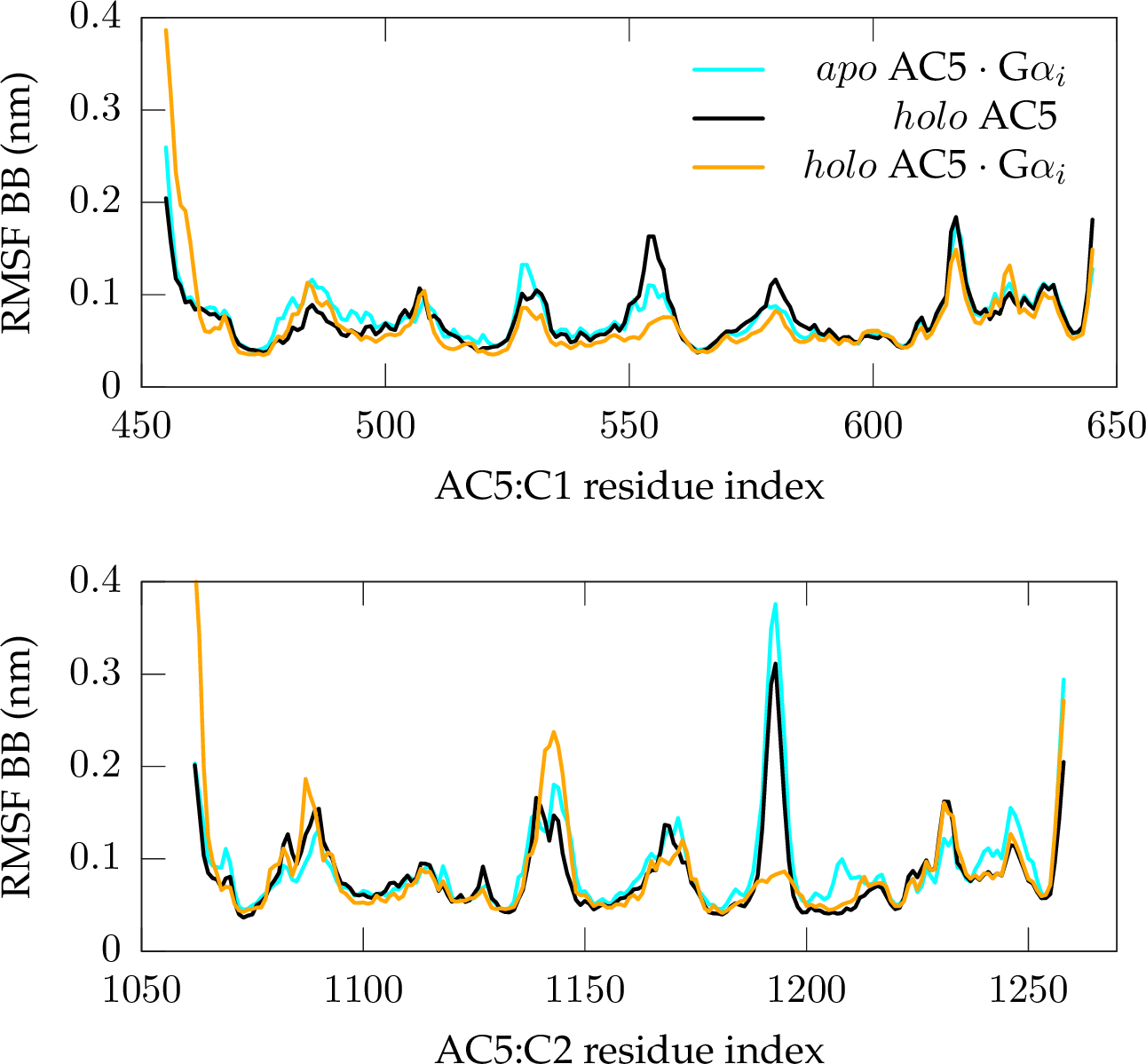

Supplement: S5 Fig — Root-mean-square fluctuations (RMSFs) of the C1 (top panel) and C2 (bottom panel) domains calculated on the protein backbone for both simulated systems, holo Gα-free and AC5:Gαi1, excluding the first microsecond of simulation and block-averaged over 100 ns segments. RMSFs of the apo AC5:Gαi1 system are also reported in cyan. (TIF) [file pone.0245197.s005.tif]

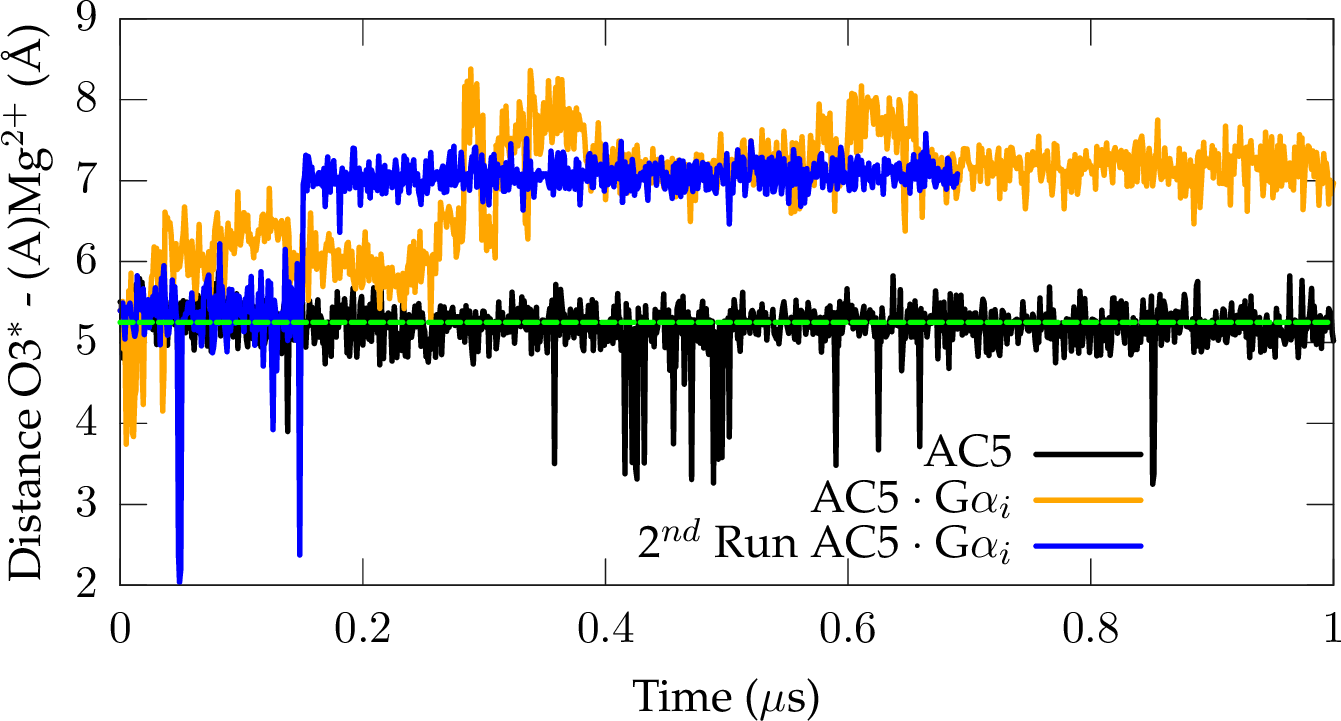

Supplement: S6 Fig — The distance between ATP’s ribosyl oxygen O3* and the neighbouring MgA2+ is reported as function of time in the first μs of simulation of holo free AC5 and holo AC5:Gαi1 complex. In the case of holo AC5:Gαi1 complex the same analysis was repeated on a second independent trajectory (blue line). The starting distance corresponding to the value found in the X-ray structure (PDB ID: 1CJK) is shown by a green dashed line. (TIF) [file pone.0245197.s006.tif]

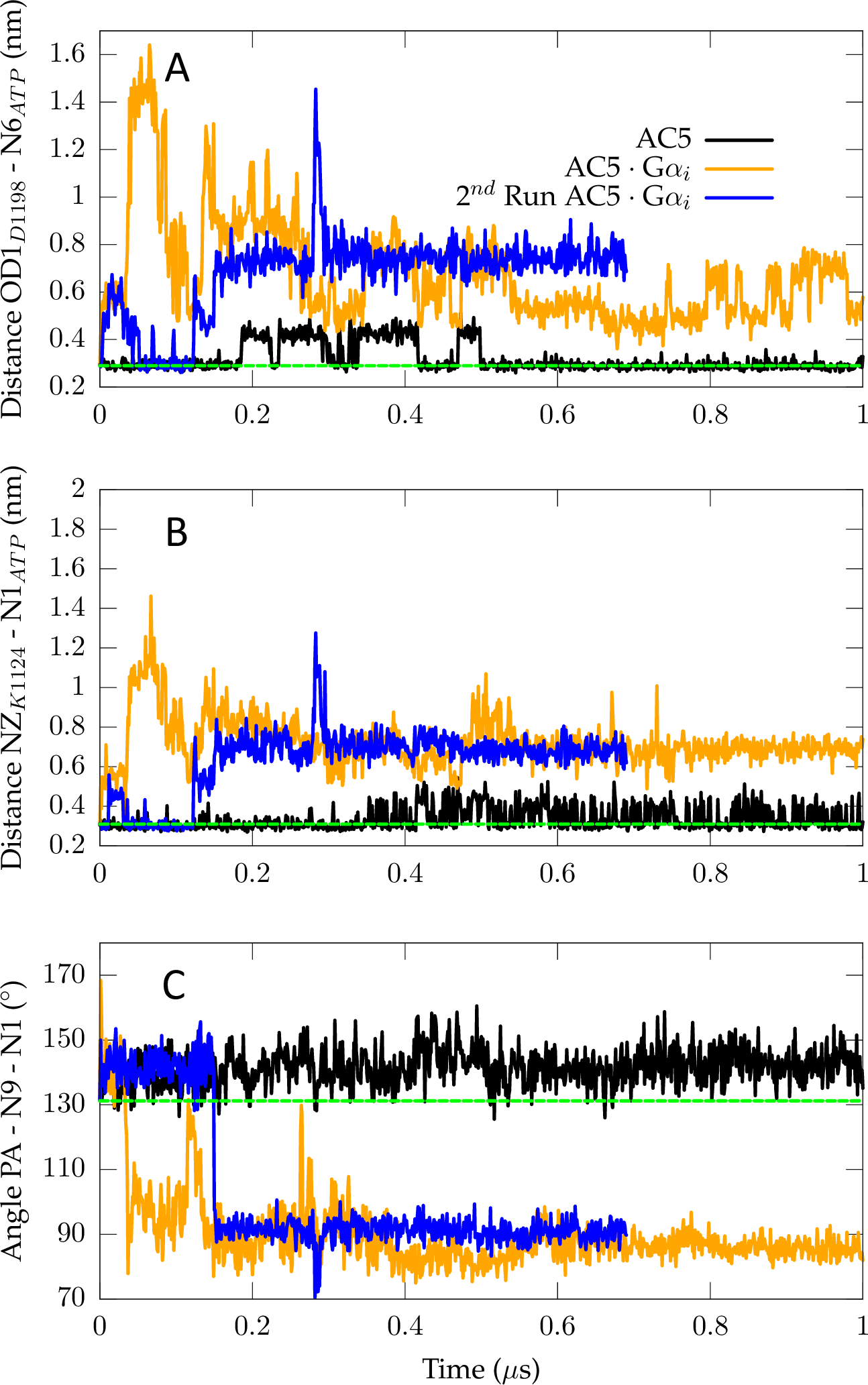

Supplement: S7 Fig — Distances between the adenine moiety of ATP and D1198 (A), D1198(OD1)-ATP(N6), as well as K1124 (B), K1124(NZ)-ATP(N1), are shown for the two simulations performed for the AC5:Gαi1 system (blue and orange) and the Gα-free AC5 trajectory (black). The dashed green line indicates the value for each initial distance in the AC5:Gαi1 and Gα-free AC5 systems. (C) is a representation of an angle between ATP’s Pα and two nitrogens in the adenine group, ATP(PA-N9-N1), which are highlighted in Fig 6. This plot depicts the two simulations performed for the AC5:Gαi1 system (blue and orange) and the Gα-free AC5 trajectory (black). The dashed green line indicates the value of the initial angle in the AC5:Gαi1 and Gα-free AC5 systems. (TIF) [file pone.0245197.s007.tif]

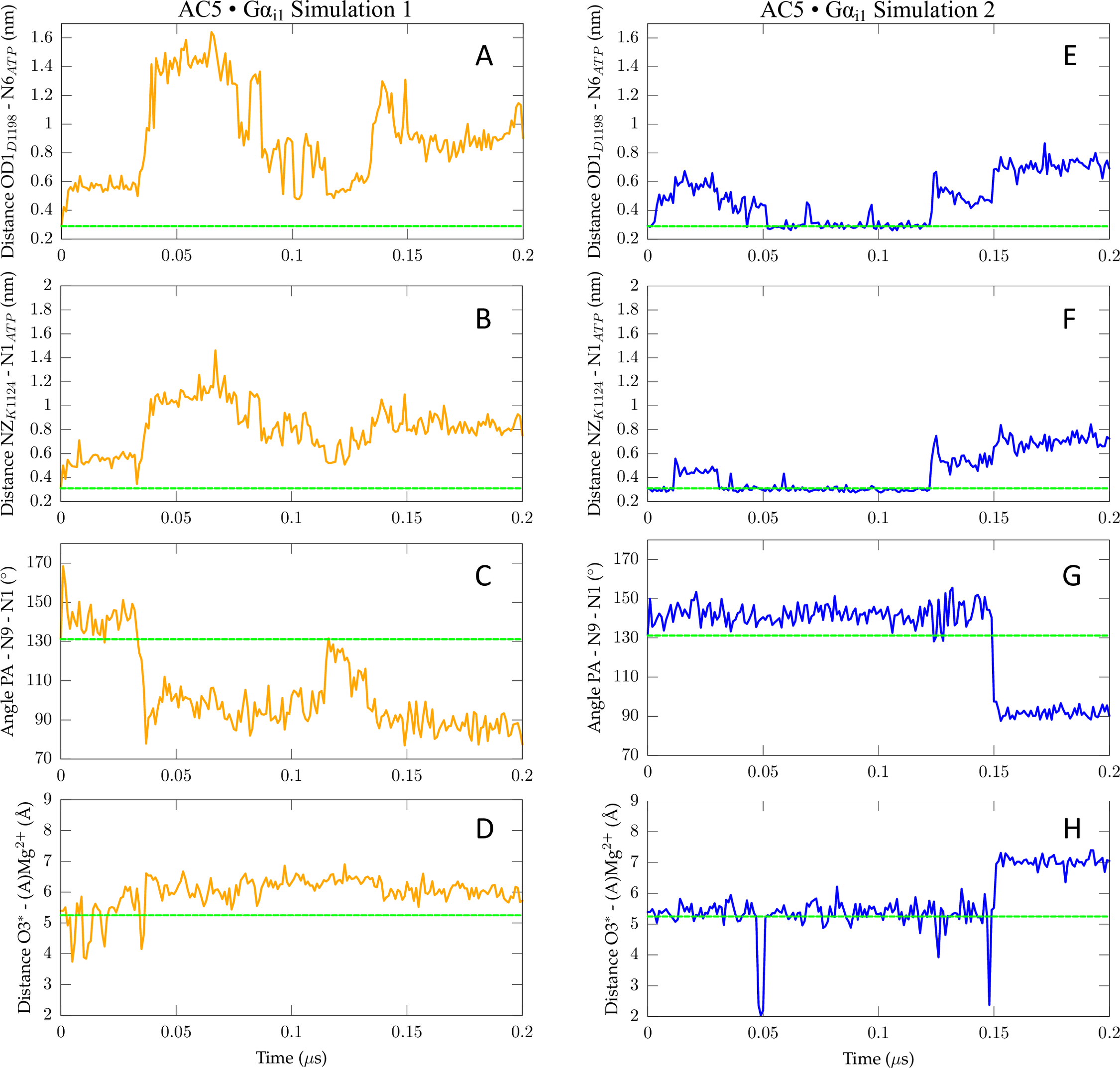

Supplement: S8 Fig — Distances between the adenine moiety of ATP and D1198 (A), D1198(OD1)-ATP(N6), as well as K1124 (B), K1124(NZ)-ATP(N1), are shown for the AC5:Gαi1 system for the first 200 ns of the trajectory. The dashed green line indicates the value for each initial distance in the AC5:Gαi1 system. The complete trajectory is represented in Fig 6. (C) Angle between ATP’s Pα and two nitrogens in the adenine group, ATP(PA-N9-N1), is shown of the first 200 ns of the AC5:Gαi1 simulation reported in Fig 6. The dashed green line indicates the value of the initial angle in the AC5:Gαi1 and Gα-free AC5 systems. (D) Distance between ATP’s ribosyl oxygen O3* and the neighbouring MgA2+ reported as function of time for the first 200 ns of simulation in the AC5:Gαi1 system. The complete trajectory is represented in Fig 6. The starting distance corresponding to the value found in the X-ray structure (PDB ID: 1CJK) is shown by a green dashed line. (E,F,G,H) represent the respective values reported in (A,B,C,D) obtained in the second run of the AC5:Gαi1 system, which was simulated for ∼0.7μs in total (S7 Fig). (TIF) [file pone.0245197.s008.tif]
